# The Mediating Effect of Self-Efficacy in the Connections between Strength-Based Parenting, Happiness and Psychological Distress in Teens

**DOI:** 10.3389/fpsyg.2017.01707

**Published:** 2017-10-10

**Authors:** Daniel J. Loton, Lea E. Waters

**Affiliations:** Centre for Positive Psychology, Melbourne Graduate School of Education, The University of Melbourne, Melbourne, VIC, Australia

**Keywords:** strength-based parenting, self-efficacy, subjective wellbeing, distress, happiness, adolescence

## Abstract

Preliminary studies of s*trength-based parenting* (SBP), a style of parenting that seeks to build strengths knowledge and strengths use in one’s child, have reported benefits such as higher life satisfaction, subjective wellbeing, and positive emotions together with lower stress in children and teens. Two proximal mediators conveying these effects have been identified: teen’s own use of strengths and strength-based coping, along with a small moderating effect of growth mindsets relating to strengths. The current study tests the potential mediating effect of self-efficacy, a sense of agency in life, in the relationship between SBP and mental health (wellbeing and illbeing) in teens. Self efficacy has been linked to wellbeing and strengths processes in past studies and is classed as a basic human need and form of eudaimonic happiness. This study reconfirmed the adaptive benefits of SBP in a large sample of Australian adolescents (*N* = 11,368; 59% male; *M*_age_ = 14.04, *SD*_age_ = 1.99) sourced from 28 schools. Using structural equation modeling, SBP significantly and directly predicted higher happiness and lower depression, with direct effects falling into the 85th and 95th percentile of meta-analytically derived individual differences effect sizes. In addition, self-efficacy was a significant partial mediator, accounting for 40.0% of the total effect on happiness and 52.7% of the total effect on distress. Self-efficacy was also a full mediator in the case of anxiety, with a strong indirect effect. Results suggest that building strengths in teens can also build self-efficacy, and given the large effect sizes, that SBP is a promising leverage point for increasing teen wellbeing.

## Introduction

“Inherently, each one of us has the substance within to achieve whatever our goals and dreams define. What is different for each of us is the training, education, knowledge and insight to utilize what we already have.”

– Mark Twain

The increased rates of mental illness in the teen years, coupled with heightened neuroplasticity and rapid psychosocial change, mean that adolescence is now recognized as a critical developmental period requiring intervention to buffer against distress and build the capacity to live well (Schwartz et al., 2012). Parenting style is a significant predictor of youth mental health during adolescence; and, indeed, for many decades later (Flouri, 2004; Huppert et al., 2010; Di Stefano and Cyr, 2014; Stafford et al., 2016). Studies of a new parenting style focussed on strengths – strength-based parenting (SBP) – have found that it has a significant, positive relationship to wellbeing in teens, partly through building teen’s own strengths knowledge and use (Waters, 2015b; Jach et al., 2017). Self-efficacy, theoretically related to strengths, has also predicted wellbeing in prior studies and represents a promising mediator in the connections between SBP, wellbeing and distress (Caprara et al., 2006). The aim of this study, therefore, is to confirm whether SBP is positively associated with wellbeing (happiness), and negatively associated with illbeing (depression, anxiety, and stress), in a large sample of adolescents; and test whether these relationships are mediated by self-efficacy.

### Teen Mental Health (Wellbeing and Illbeing)

Adolescence is a life stage marked by identity formation and individuation, widening social bonds, as well as heightened neurological and dispositional plasticity. The teen years are therefore increasingly recognized as a key formative period for positive assets that underpin wellbeing (Sawyer et al., 2012; Patton et al., 2016). For a substantial number, adolescence is also characterized by the first appearance of mental illness, coupled with a decrease in life satisfaction, where several factors converge to produce what can be a difficult and potentially pivotal time for health outcomes (Cimpian et al., 2007; Kessler et al., 2007; Andersen and Teicher, 2008). According to a recent national survey in Australia, the 12-month prevalence of mental disorders in 11–17 year-olds was 14.3%, with a notable shift from attention-regulation and conduct disorders in children, to the potentially more serious conditions of anxiety and depression in teens (Lawrence et al., 2016). Suicide is now the leading cause of death for young and middle-aged Australians (Australian Bureau of Statistics [ABS], 2015). Global rates are similar, with the World Health Organization [WHO] (2011) estimating mental illness prevalence at 13%, with unipolar depression already the third leading cause of global disease burden. These figure are likely to rise. Global health projection studies herald a pivot from communicable diseases, with the exception of HIV in developing countries, to lifestyle-related factors, chronic conditions and mental illness as the driving forces behind morbidity and mortality (Mathers and Loncar, 2006; Patton et al., 2016).

The consequences of ignoring mental illness at youth are especially critical. Mental illness is often associated with impaired functioning, which in youth can pose threats to normal development. Mental illness in teens significantly predicts days missed at school (Lawrence et al., 2016), which may adversely affect academic and occupational futures, and in-turn, later socioeconomic resources; a potentially compounding and negative reinforcing spiral. Thus it is imperative to understand the factors and processes that can prevent or buffer against mental illness during adolescence.

Yet to focus only on mental illness in teens would be to disregard the important findings coming from positive psychology that mental health is more than the absence of mental illness and that it also includes the presence of mental wellness (Keyes, 2002). These findings tell us that building a full state of mental health in teens involves understanding the factors that minimize illbeing *and* studying the factors that maximize wellbeing, the latter of which is defined as a state of feeling good and functioning well (Huppert and So, 2013). Past research on happiness and subjective wellbeing has shown that they are distinct from, and potentially preventative of, mental illness (Veenhoven, 2008; Wong, 2011; Hoyt et al., 2012). Therefore, the current paper includes indicators of what may be termed the negative end of the mental health spectrum (depression, stress and anxiety) *and* indicators of the positive end of the mental health spectrum (happiness).

Following Kashdan et al.’s (2008) review of how happiness is construed in contemporary psychological studies, we define happiness as the ability to experience frequent positive emotions and a subjective satisfaction with life^1^. While the determinants of happiness and SWB have now received extensive study in adults, studies of these states in youth are still growing (Proctor et al., 2009). In terms of a simple overall snapshot, recent global studies indicate around 60% of youth are happy with their lives, showing much room for improvement (Helliwell et al., 2016; Broadbent et al., 2017). Meta-analytic studies complement these findings, indicating happiness is predicted by educational attainment and cognitive ability (Witter et al., 1984; Harris et al., 2016), socio-economic status and various forms of social support (Pinquart and Sörensen, 2000), physical health (Veenhoven, 2008), personality traits (Harris et al., 2016) and a connectedness with nature (Capaldi et al., 2014). In the current study we explore the role of parenting, specifically SBP, in predicting teen happiness.

### Strengths and Teen Mental Health

Strengths are defined here as natural capacities that are experienced as energizing, authentic, and virtuous (Govindji and Linley, 2007). They can be character strengths (i.e., strengths of personality) or abilities and talents. Attesting to their importance in unlocking human potential and wellbeing, Seligman and Csikszentmihalyi (2000, p. 8) called for “massive research in human strengths” in their foundational paper launching the field of positive psychology.

Several studies have connected strengths with wellbeing in children and teens. In a qualitative study with 680 parents of children between the ages of 3 and 9, parents’ written descriptions of strengths in their children were found to be related to child SWB (Park and Peterson, 2006). Strengths have been shown to help first-graders transition smoothly into elementary school (Shoshani and Aviv, 2012). In a sample of 10–11 year olds, using strengths to cope with challenge was found to reduce stress (Waters, 2015a). In 12–13 year olds, strengths assisted in transition to middle school and predicted life satisfaction, positive affect and negative affect (Shoshani and Slone, 2013). Studies with teenagers and young adults have found that using strengths is related to life satisfaction, wellbeing, self-efficacy, self-esteem, and positive emotions (Proctor et al., 2011b; Allan and Duffy, 2014; Suldo et al., 2014; Douglass and Duffy, 2015; Jach et al., 2017). Importantly, strengths in young adolescents have also demonstrated malleability. In a strengths intervention, Proctor et al. (2011a) reported that 12–14 year olds who completed 3–12 strengths lessons reported a significant increase in strengths use and in-turn, life satisfaction, relative to adolescents who did not participate in the exercises.

While there are fewer studies on the strengths-illbeing link, some studies have tied strengths with a reduction in mental illness in youth. Gillham et al. (2011) studied the role of strengths in the transition of students from Year 9 to Year 10 in American schools. The authors found that prosocial strengths predicted fewer depression symptoms after the transition to Year 10. Some studies position strengths as a preventative factor against development of mental illness. In a representative community sample of 688 mothers from upstate New York, it was found that youth with multiple personality strengths at age 16 were significantly less likely to develop subsequent psychiatric disorders at age 22, even after accounting for the effect of demographics, socioeconomic status, intelligence and baseline levels of mental illness (Bromley et al., 2006). In intervention studies that target building and reflecting on strengths, studies have found significant decreases in depression symptoms for participants who adhered to the intervention protocols for a period of time (6 months); see Seligman et al. (2005) for a review and Gander et al. (2013) for a replication.

### Parenting and Teen Mental Health

A multitude of studies connect parenting with teen wellbeing, although with varying effect sizes (Steinberg, 2001). Researchers have suggested multiple pathways by which parenting can influence child/teen wellbeing including socio-relational, psychological and socio-economic; the primary mechanisms considered to be parental attachment, parental involvement and parental style (Stafford et al., 2016). For example, a recent meta-analysis examined the effect of parental attachment, defined as perceptions of safety and stability in the parent-adolescent relationship, on the wellbeing of late adolescents during their adolescence-to-adulthood transition in college students (Mattanah et al., 2011). Across 156 suitable studies and *N* = 32,969 participants, the average effect of parental attachment on positive development at college was moderate and significant (*r* = 0.23), for both mother and father involvement. In addition, the authors analyzed effect size distributions across several different outcome domains, with the average effect size of parental attachment on negative emotions being inverse (*r* = -0.21). McLeod et al. (2007b) undertook a meta-analysis of 47 studies examining the effect of parenting on child anxiety, and concluded that parenting overall explained only 4% of variance in anxiety; but the specific parental sub-dimension of autonomy-granting upward of 18%. The same authors also examined meta-analytically derived estimates of the effect of parenting on depression, and concluded that a marginally higher amount of 8% of youth depression was accounted for by parenting (McLeod et al., 2007a).

Several studies suggest these effects may be lifelong. In one longitudinal study with a nationally representative sample from the United Kingdom (*N* = 11,419), Flouri (2004) assessed the effect of several family variables, including parental involvement/closeness, measured in childhood and again at adolescence, in predicting SWB in mid-adulthood. Perceived closeness to mother at age 16 predicted life satisfaction at age 42, for both men and women. In addition, parenting factors had indirect effects on subsequent SWB through protecting against psychological illbeing, promoting educational attainment and increasing the odds of finding a partner. A further longitudinal study of 984 women from the 1946 British birth cohort study found that recollections of parental style at age 43 predicted psychological wellbeing 9 years later (Huppert et al., 2010). In this study three distinct parental styles were identified: care, non-engagement and control, as measured by the Parental Bonding Instrument, that in-turn predicted higher levels of Ryff’s psychological wellbeing scale. Parental care was associated with higher wellbeing, while parental non-engagement and control with lower wellbeing. In Canada, a longitudinal study of a nationally representative sample of youth found that parental quality, a composite of positive parent–child interactions, consistent discipline and autonomy-granting, completely mediated the negative effects of divorce on subsequent child wellbeing, after accounting for other sociodemographic factors (Di Stefano and Cyr, 2014).

Clearly, parenting has an impact on teen mental health and the years beyond, and is therefore an important factor to study. However there are gaps in knowledge on parenting style and wellbeing. Researchers note a preponderance of studies on the caring and control dimensions of parenting, with fewer focussed on building positive capacities such as strengths (Mattanah et al., 2011; Williams et al., 2012). Given the long-lasting effects of parenting in the teenager years, it is important to explore potential process variables that may convey the effect of parental styles on theorized health and wellbeing outcomes, including self-efficacy.

### SBP and Teen Wellbeing

Strength-based parenting is a newly created construct that fuses strengths processes with parental style (Waters, 2015a; Waters and Sun, 2016). It is defined as “a style of parenting that seeks to deliberately identify and cultivate positive states, positive processes and positive qualities in one’s children” (Waters, 2015a) and includes a focus on the parents themselves knowing and deploying their own strengths in their role as a parent (Waters and Sun, 2016). Waters (2015a,b) aligned SBP with Govindji and Linley’s (2007) two factor model of strengths: strengths knowledge and strengths use. *Strength knowledge* is defined as a person’s “awareness and recognition of their strengths” (Govindji and Linley, 2007, p. 147), whereas *strengths use* is defined as the extent to which individuals “use their strengths in a variety of settings.” Subsequent psychometric analyses found that SBP is comprised of these two highly correlated, but unique, factors and that strength-based parents are those who understand the child’s strengths and encourage the child to use his/her strengths (Jach et al., 2017). Dyadic studies show that child and parent ratings of these two factors in each other converge to a moderate degree (Waters, 2015b).

A research program on SBP has reported numerous adaptive relationships between SBP with children and adolescent wellbeing. For example, children of strength-based parents tend to have lower stress levels, a relationship partly bridged by their use of strength-based coping (Waters, 2015a). In teenage samples, SBP predicts life satisfaction over and above other adaptive parental styles, namely authoritative parenting (Waters, 2015b), and this prediction remains significant over time. In addition, SBP predicts teenagers own awareness and use of their strengths (Waters, 2015b), engagement, persistence and academic grades (Waters et al., in press). Interestingly, the relationship between SBP and teen strength use is moderated, to a small degree, by growth mindsets (Jach et al., 2017), suggesting that those teens who are open to growth and change will benefit more from having strength-based parents. Parents, themselves, also benefit from SBP. A brief intervention was undertaken aiming to increase strengths-based approaches with a small group of parents, with an evaluation finding increased parental self-efficacy and positive affect post-intervention compared with a waitlist comparison group (Waters and Sun, 2016).

Theoretical mechanisms behind the benefits of SBP for both the parents and children/teenagers are hypothesized to operate intrapersonally, through building a positive identity, and interpersonally, through building positive parent–child relationships; as well as increased social bonds in the case of prosocial strengths. With respect to identity, Waters (2015a) argued that SBP creates a positive filter for a child’s identity as they see themselves through the lens of their strengths. With respect to parents’ identity, Waters and Sun (2016) suggested that SBP helps to foster an identity of a competent parent. Interpersonally, SBP, by providing an atmosphere that regularly supports the development and reinforcement of strengths within the family, is likely to build mutual regard and respect. In the Waters and Sun parent intervention, parents were taught the process of strength spotting which has been found, in other studies, to heighten affiliative motivation, positive communication and having warm, reliable interpersonal relationships (Komazawa and Ishimura, 2015). Indeed, researchers at the Department of Clinical Psychology at Tokyo Seitoku University state that “People who are good at spotting others’ strengths seem to build warm, trustworthy, and positive relationships” (Komazawa and Ishimura, 2015).

### Self-Efficacy and Wellbeing

Past studies have found that self-efficacy is also a determinant of teen wellbeing (Caprara et al., 2006), and has been classified as a basic human need (Ryan and Deci, 2000; Bandura, 2008). Self-efficacy is a general sense of one’s competence and ability to fulfill goals in life (Schwarzer and Jerusalem, 1995; Zimmerman, 2000). Like other components of wellbeing, self-efficacy and associated causal and control beliefs are variously placed under the banners of non-cognitive, social-emotional, soft, character and positive education skills (Durlak et al., 2011; Waters, 2011). Several prominent theories of optimal human functioning, including self-determination theory (SDT) and social-cognitive theory (SCT), place a sense of agency as a basic human need (Ryan and Deci, 2000; Bandura, 2008). In a recent study of life satisfaction in 437 Swiss adolescents, self-efficacy was a significant predictor of life satisfaction, even after accounting for personality and self-esteem (Marcionetti and Rossier, 2016). In a German study of university students (*N* = 180), general self-efficacy was found to mediate the well-established connection between personality and SWB (Strobel et al., 2011).

In a longitudinal study of Italian adolescents (*N* = 664), Caprara et al. (2006) found that affective and interpersonal self-efficacy were robust predictors of happiness both concurrently and 2 years later. A further study of Italian adolescents (*N* = 650) supported these findings, reporting academic and social self-efficacy beliefs were stronger predictors of subsequent life satisfaction than peer preference or prior academic performance (Vecchio et al., 2007). In a study with a small but unique cohort of children attending a mid-western summer camp in the United States for children with chronic illness or disability (*N* = 53), it was found general self-efficacy beliefs predicted lower anxiety levels, and this connection was mediated by self-esteem (Dahlbeck and Lightsey, 2008).

### Self-Efficacy and Parenting

There are relatively few studies directly investigating the role of parents in building the general self-efficacy of their children (Mattanah et al., 2011); but some suggest long-lasting effects. In a cross-sectional study of university students (*n* = 186), recollection of earlier unfavorable parental rearing style (lacking affection but controlling) predicted a marginal amount of variance in self-reported general self-efficacy (Oliver and Paull, 1995). In the same study, unfavorable parental rearing style also predicted a small amount of variance in depression symptoms; around 13% in both variables. In a longitudinal study of 984 women from the United Kingdom, recollections of earlier parental style predicted Ryff’s definition of psychological wellbeing (PWB) at age 52 (Huppert et al., 2010). Importantly, Ryff’s model includes a sub-scale of ‘environmental mastery,’ which is a close proximate of general self-efficacy, with some distinctions (Ryff and Singer, 2013). In that study, women raised by parents who had a controlling style showed a direct inverse connection with PWB decades later.

### Self-Efficacy as a Potential Mediator

There are clear theoretical links between self-efficacy and strengths. Some studies have evaluated the relative utility of strengths as a predictor of happiness, alongside self-efficacy. Proctor et al. (2011a) examined relationships between SWB, the Values in Action (VIA) strengths taxonomy and self-efficacy in a sample of college students (*N* = 135), finding that hope and zest significantly (*p* < 0.05) predicted life-satisfaction. The authors also reported a large correlation between strengths use and self-efficacy, as well as significant and unique predictive utility of both variables in a regression model predicting SWB; but did not conduct a formal test of mediation. In another study of college students (*N* = 214), both SWB and PWB correlated strongly with general self-efficacy and strengths use; as well as vitality and self-concordant goals (Govindji and Linley, 2007). Yet in this study, self-efficacy diminished to non-significance when included in a regression model alongside other psychological predictors. However, strengths use remained moderate and significant.

In a study of Israeli adolescents (*N* = 396) also using the VIA taxonomy, strengths explained 46% of the variance in general self-efficacy, and 32% of life satisfaction scores, with leadership strengths showing the largest relationship (Weber et al., 2013). The authors then tested a mediation model, finding evidence that general self-efficacy is a ‘full’ mediator of the connection between leadership strengths and life satisfaction. It is currently unclear whether self-efficacy will mediate the relationship between more general strengths processes (i.e., not leadership-specific) and SWB, and also whether the promotion of self-efficacy may explain the connections between parental styles that promote strengths processes, and teen wellbeing.

### Summary

In summary, parental style is an important factor that predicts the wellbeing of teens and can impact life trajectory. While most parent–child research is focused on the caring and control dimensions of parental style (Mattanah et al., 2011; Williams et al., 2012), the current study forms part of a new way of understanding parenting style, by adopting a positive psychology approach and investigating the effects of SBP. Strengths and self-efficacy have both predicted wellbeing in youth, and mixed findings have been reported regarding self-efficacy as a potential mediator of the strengths-wellbeing connection. Accordingly, this study seeks to confirm relationships between SBP and mental health in teens; and also test whether self-efficacy explains some of this relationship. Cognisant of the assertion that to look exclusively at either illbeing *or* wellbeing is to miss half of the picture (Wong, 2011), this study has included indicators of both, namely depression, anxiety and stress; and happiness (comprised of positive emotion and satisfaction with life).

**FIGURE 1:**
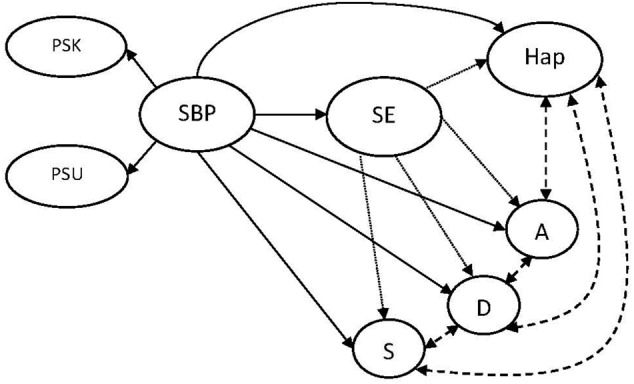
The proposed structural equation model. Solid lines denote regression weights and dashed lines denote correlations. PSK, strength-based parenting knowledge component; PSU, strength-based parenting use component; SBP, strength-based parenting; SE, self-efficacy; Hap, happiness; A, anxiety; D, depression; S, stress.

In teens, we hypothesize the following:

(1) Higher SBP will predict lower depression, anxiety and stress, and higher happiness.
(2) Self-efficacy will be related to SBP, distress and happiness, satisfying the general preconditions for mediation.
(3) SBP will demonstrate significant direct effects, and indirect effects via self-efficacy, on distress factors (inverse relationships) and happiness (a positive relationship); a partial mediation (**Figure 1**).

## Materials and Methods

### Participants and Procedure

A large sample of students (*N* = 11,138) were drawn from 28 schools across 2 states (Victoria; New South Wales) in Australia. The mean age of students was 14.04 (*SD*_age_ = 1.99; range 10–18, *n* = 37 did not indicate an age) and 59% of the sample were male. Items in the current study were drawn from a larger comprehensive wellbeing survey, known as the Wellbeing Profiler, that was developed by a team of researchers at the Centre for Positive Psychology, University of Melbourne^2^. All recruitment and procedures in this study complied with the National Statement on Ethical Conduct in Human Research and were approved by the University’s Human Research Ethics Committee^3^.

Teachers invited students to complete an online survey in-class. In addition to Principal, School and parental consent, students were also offered assent in completing the survey (i.e., they were provided information about the project and offered the choice of completing the survey or alternative, educationally appropriate activities assigned by their teacher). All Schools were provided with a report detailing aggregate, year-level wellbeing metrics. Student-level data was not provided to schools, and all identifying information about schools and students was removed from the larger, Wellbeing Profiler dataset prior to extraction of the items for the current study. As such, while it is highly likely that schools in the sample differed substantially from one another on a variety of characteristics such as socio-economic status and academic performance, we were unable to asses these factors in the current study due to the strict ethical requirements in handling the larger Wellbeing Profiler data set.

The nature of the procedure and data in this study share some characteristics with secondary analyses. Firstly, brevity is a concern due to the multiple constructs included in the larger Wellbeing Profiler project and the burden this places on participants. As such, the authors of the larger Wellbeing Profiler study chose to include fewer indicators per construct in order to facilitate wider inclusion of a range of mental health indicators. Given that this is a common approach when collecting data from large sample sizes (McHorney et al., 1994), statisticians have considered the trade-off between reliability of briefer measures and the inclusion of more latent factors. Where appropriate, such as in large surveys, some argue that more latent factors facilitate testing of interactions and confounding effects, offering more research utility than fewer factors with higher measurement reliability (see Hayduk and Littvay, 2012 for a statistical consideration and subsequent debate). Indeed, studies have compared the relative utility of long and short form health scales, concluding that there is value in even single-indicator scales (McHorney et al., 1994), which are used in population health studies. All scales used in the current study had more than two indicators, with the exception of distress (which had two items for depression, two items for anxiety and two items for stress)^4^.

### Materials

#### Strength-Based Parenting

Due to space limitations in the survey, an abridged version of the SBP scale that was first developed by Waters (2015a,b) and later refined by Jach et al. (2017) was used with the current sample. Following Govindji and Linley’s (2007) two factor model of strengths, students completed a scale that assessed the degree to which their parents know their strengths [SBP-Knowledge; three items; e.g., *My parent/carer(s) knows the things I am good at*] and the degree to which they feel their parents encourage them to use their strengths [SBP-Use; three items; e.g., *My parent/carer(s) give me lots of opportunities to use my strengths*]. The six SBP items were anchored on a 7-point scale from *strongly disagree* to *strongly agree*. Reliability of this measure has been shown to be strong in other youth samples in past studies (Waters, 2015a,b; Jach et al., 2017) and remained strong in this sample with the reduction in study items (SBP-Knowledge ω = 0.89, 95% CI [0.89, 0.90]; SBP-Use ω = 0.83, 95% CI [0.83, 0.84]).

#### Self-Efficacy

A 3-item measure of self-efficacy was adapted from the Schwarzer and Jerusalem (1995) 10-item General Self-Efficacy scale, with the phrasing of the second and third item altered to be more developmentally appropriate. The items assessed the adolescent’s judgments of their ability to act and perform at a sufficient level to attain desired end goals (Zimmerman, 2000), and included:

*I can usually handle whatever comes my way, I can solve most problems if I make the necessary effort*, and *I am able to deal with unexpected events*, anchored on a 7-point scale from *strongly disagree* to *strongly agree* (ω = 0.76, 95% CI [0.75, 0.77].

#### Happiness

The happiness sub-scale of the EPOCH measure of adolescent wellbeing developed by Kern et al. (2016) was used. This 4-item measure assesses: (a) a steady state of positive mood (e.g., *I am a cheerful person*) and b) being content with one’s life (e.g., *I have a lot of fun*). The items were anchored on a 7-point scale from *strongly disagree* to *strongly agree*. Kern et al. (2016) found strong reliability in their samples (α = 0.86), as well as good test–retest reliability and evidence of convergent and divergent validity. The current sample had an ω reliability co-efficient of 0.89 (95% CI [0.89, 0.90]).

#### Distress

An abridged version of the DASS (Depression Anxiety Stress Scale) by Lovibond and Lovibond (1995) was adapted to be more developmentally appropriate. Two-items measured each construct (six items in total) for anxiety (e.g., *I often feel nervous or anxious;* ω = 0.78, 95% CI [0.77, 0.80]), depression (e.g., *I often feel sad or hopeless;* ω = 0.81, 95% CI [0.80, 0.82]), and stress (e.g., *I often feel like I’m losing control over my life;* ω = 0.51, 95% CI [0.51, 0.55]). For parsimony the researchers initially modeled distress as a single higher order factor, but psychometric validation of the DASS indicates that depression, anxiety and stress are distinct constructs (Lovibond and Lovibond, 1995). As such, modeling all three sub-constructs as a single higher order factor may mask more nuanced relationships between predictors (in this case SBP and self-efficacy) and each distress factor. As such, stress, anxiety and depression were modeled separately in the final model. Consistent with Lovibond and Lovibond’s validation work, the use of separate factors did reveal unique relationships between anxiety and depression (and is adopted as the final model).

#### Analysis Plan

Data cleaning was undertaken using SPSS and Excel. Descriptive and inferential analyses were undertaken using R (version 3.3.1). Within R, the lavaan package (Rosseel, 2012) was used for structural equation modeling (SEM). For the mediation analysis, we used 10,000 bootstrapped standard errors, as recommended by Hayes (2009), with 95% confidence intervals reported below. Multi-group analysis examining gender differences was undertaken using MPlus Version 7.

## Results

### Descriptive Statistics

Means, standard deviations, number of cases, skew and kurtosis, and correlation matrix for all variables of interest were calculated and are presented on **Table 1**. Skew and kurtosis values were within acceptable ranges for all variables (Hair et al., 2010). As **Table 1** indicates, all correlations were statistically significant even with Holm-Bonferroni correction for multiple comparisons. In particular, SBP had substantial correlations with both self-efficacy (*r* = 0.51), and happiness (*r* = 0.60), in addition to negative correlations with distress variables (anxiety *r* = -0.22, depression *r* = -0.43, and stress *r* = -0.31).

**Table 1:** Descriptive statistics and zero-order correlations of observed variables.

Variable	*M*	*SD*	*N*	Skew	Kurtosis	1	2	3	4	5
(1) SBP	5.73	1.18	11138	–1.19	1.46					
(2) Self-efficacy	5.27	1.09	9958	–0.63	0.43	0.51				
(3) Happiness	5.69	1.22	11138	–1.29	1.55	0.60	0.56			
(4) Anxiety	4.20	1.78	11138	–0.11	–1.02	–0.22	–0.34	–0.40		
(5) Depression	2.97	1.65	11138	0.62	–0.56	–0.43	–0.47	–0.67	0.57	
(6) Stress	3.88	1.60	11138	0.06	–0.78	–0.31	–0.42	–0.47	0.70	0.62

*SBP, strength-based parenting; efficacy, self-efficacy; esteem, self-esteem. All correlations presented here are statistically significant at *p* < 0.001 (Holm-Bonferroni-corrected for multiple comparisons).*

### Structural Equation Modeling

#### Measurement Model

To follow the two-step approach to SEM advocated by Anderson and Gerbing (1988), we first examined the measurement model corresponding to our hypothesized constructs prior to conducting the mediation analysis. A Confirmatory Factor Analysis (CFA) was undertaken in which each of the latent variables were allowed to freely correlate with one another. The constructs included in the CFA were self-efficacy, happiness, anxiety, depression, stress and SBP (considered a second-order latent factor comprised of SBP-Knowledge and SBP-Use). Hu and Bentler (1999) suggest benchmarks of Comparative Fix Index (CFI) ≥ 0.95, Root Mean Square Error of Approximation (RMSEA) ≤ 0.06, and Standardized Root Mean Square Residual (SRMR) ≤ 0.08 to describe a good model fit. Our model was a reasonably good fit to the data according to these rules of thumb, χ^2^(135) = 7004.473, *p* < 0.001, CFI = 0.946, RMSEA = 0.068, and SRMR = 0.056. Factor loadings were high for all indicators, with the lowest standardized loading of 0.650 for a self-efficacy item, and most other larger than 0.8. However, the *stress* variable demonstrated poor reliability in our sample, with an omega reliability coefficient substantially lower than the other scales (stress ω = 0.51; the next lowest omega reliability was self-efficacy, ω = 0.76). We investigated the measurement model without the addition of this variable. The model provided a better fit, χ^2^(107) = 3127.839, *p* < 0.001, CFI = 0.968, RMSEA = 0.050, and SRMR = 0.035. Due to this superior fit, the poor reliability of the measure and prior research having investigated the SBP-teen stress link (Waters, 2015a), we decided to remove the stress variable in our subsequent mediation analysis.

#### Mediation Model

**FIGURE 2:**
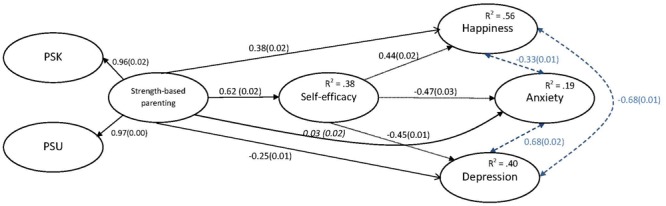
The full mediation model. Values depict standardized β weights, and values in parentheses depict the standard error of β. For convenience indirect effects are denoted by dotted lines and correlations by dashed lines. Non-significant (*p* > 0.05) pathways are denoted in italics. Indicators of the latent factors are not displayed for brevity. PSK, parental strength knowledge; PSU, parental strength use.

A mediation analysis using Full Estimation Maximum Likelihood was undertaken to investigate the model outlined in **Figure 1**, in which self-efficacy was proposed to mediate the relationship between SBP and both happiness and distress. The mediation analyses was a very good fit to data across the different fit indices, χ^2^(107) = 2057.715, *p* < 0.001, CFI = 0.982, RMSEA = 0.040, SRMR = 0.024; and the model explained 56% of the variance in happiness, 40% of the variance in depression and a smaller 19% of variance in anxiety (**Figure 2**). The mediation analysis revealed a significant indirect effect of SBP via self-efficacy on all outcome variables. SBP significantly predicted higher happiness, via self-efficacy, β = 0.27, *SE* = 0.01, *Z* = 23.10, *p* < 0.001; lower depression via self-efficacy, β = -0.28, *SE* = 0.01, *Z* = -22.83, *p* < 0.001; and lower anxiety via self-efficacy, β = -0.29, *SE* = 0.01, *Z* = -22.35, *p* < 0.001. As estimates of indirect effects can produce symptotic distributions, bootstrapping confirmed their significance. The 95% confidence intervals for the standardized indirect effects did not include 0, ranging from 0.25 to 0.28 for happiness, -0.29 to -0.24 for depression, and -0.30 to -0.25. There remained a significant direct effect of SBP on happiness, β = 0.38, *SE* = 0.02, *Z* = 24.32, *p* < 0.001, and also a significant direct effect of SBP on depression, β = -0.25, *SE* = 0.02, *Z* = -14.93, *p* < 0.001. Yet no direct effect remained for anxiety, indicating full mediation, β = -0.03, *SE* = 0.02, *Z* = 1.620, *p* < 0.001. Decomposing the effects in **Figure 2** further, and in proportionate terms the indirect effect of self-efficacy accounted for 40.9% of SBP’s total effect on happiness, and 52.7% of SBP’s total effect on depression. Parameter estimates reported here are standardized to the predictor and outcome variables.

#### Gender Differences

Multi-group analysis was used as the framework to explore potential gender differences in the mediation model depicted in **Figure 2** (see Byrne, 2012). Participants with invalid gender (*n* = 295, or 2.6%) were removed for this analysis. The measurement model was first run for males and females separately; with very similar and good fit to the data in each case: females (*n* = 4753) χ^2^(107) = 1174.002, *p* < 0.001, CFI = 0.976, RMSEA = 0.046, SRMR = 0.026; males (*n* = 6385) χ^2^(107) = 1027.690, *p* < 0.001, CFI = 0.982, RMSEA = 0.040, SRMR = 0.024.

The configural measurement model was then calculated χ^2^(214) = 2201.692, *p* < 0.001, CFI = 0.982, RMSEA = 0.041, SRMR = 0.024, and factor loading invariance then tested by constraining factor loadings to equality across males and females, which resulted in a significant decrement in fit χ^2^(225) = 2292.104, *p* < 0.001, CFI = 0.981, RMSEA = 0.041, SRMR = 0.027, Δχ^2^(11) = 90.412, *p* < 0.001.

Modification indices pertaining to the equality constrained factor loadings were examined and indicated happiness item 2 (“*I am a cheerful person*”) appeared to be interpreted differently across gender, loading more highly for females than males [M.I. = 18.87, standardized expected parameter change (S.EPC) = 0.04 for females; -0.03 for males]. After this factor loading was freely estimated model fit improved somewhat, χ^2^(224) = 2273.134, *p* < 0.001, CFI = 0.981, RMSEA = 0.041, SRMR = 0.027, Δχ^2^(1) = 17.97, *p* < 0.001. Modification indices pertaining to constrained factor loadings were again examined, and happiness item 1 (“*I have a lot of fun.*”), represented a higher loading for males than females (M.I. = 25.18, S.EPC = -0.06). After freeing this loading, fit again improved χ^2^(223) = 2247.601, *p* < 0.001, CFI = 0.981, RMSEA = 0.040, SRMR = 0.026, Δχ^2^(1) = 25.533, *p* < 0.001. Only one constrained factor loading now remained in the modification indices, self-efficacy item 2 (“*I can solve most problems if I make the necessary effort*.”), which loaded lower for females than males (M.I = 18.899, S.EPC = -0.04). After removal of this remaining factor loading constraint, fit again improved χ^2^(222) = 2227.681, *p* < 0.001, CFI = 0.982, RMSEA = 0.040, SRMR = 0.025, Δχ^2^(1) = 19.92, *p* < 0.001. Given the low impact on goodness-of-fit statistics outside of chi-square, we then proceeded to testing for structural model invariance in the final mediation model. With all factor loadings aside from the three identified above constrained to equality, the mediation model depicted in **Figure 2** was then estimated; first with the seven structural paths freely estimated, then again with them fixed to equality across males and females. This partial invariance configural mediation model produced identical fit statistics as the configural measurement model above, in which all latent factors were free to correlate.

**FIGURE 3:**
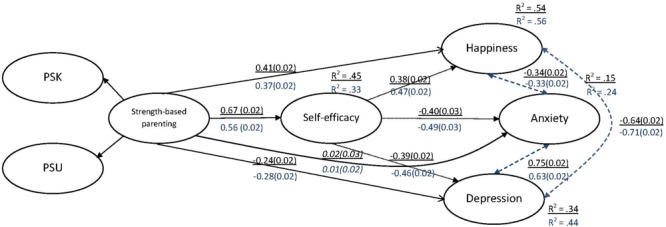
Multigroup analysis of the mediation model across gender. Simultaneously estimated, standardized β weights for the partial invariance mediation model across gender are depicted, with standard error of β in parentheses. Underlined parameters represent the estimates for females, and non-significant (*p* > 0.05) pathways are denoted in italics. For convenience indirect effects are denoted by dotted lines and correlations by dashed lines. Indicators of the latent factors are not displayed for brevity. PSK, parental strength knowledge; PSU, parental strength use.

Parameter estimates for the male and female subgroups generated in this partial invariance mediation model are presented in **Figure 3**. No major differences were evident. Variance explained in distress factors were slightly higher for males, and lower in the case of self-efficacy. SBP had a slightly smaller effect on self-efficacy for males, and self-efficacy had slightly larger effects on the outcomes, but other parameter estimates are very similar across gender. Bootstrapped confidence intervals for the key indirect effects of interest did not contain zero, confirming significance of the mediating effect in both.

Finally, fixing the structural paths to equality resulted in a significant but small decrement in overall fit χ^2^(229) = 2308.200, *p* < 0.001, CFI = 0.981, RMSEA = 0.040, SRMR = 0.030, Δχ^2^(7) = 80.52, *p* < 0.001, but specific goodness-of-fit indices moved very little: the CFI only at the third decimal point, the RMSEA identical; the SRMR only at the second decimal point. Fit statistics also exceed Hu and Bentler’s simulation-based benchmarks, indicating a good fit to the data. Resultantly, this analysis suggests the majority of scale items, and the overall mediation model, operate similarly across gender sub-groups.

## Discussion

Consistent with an increasing focus on early intervention, the teenage years are coming to be viewed as a unique window of opportunity for laying psychosocial foundations that will support lifelong health and wellbeing (Sawyer et al., 2012; Patton et al., 2016). Strengths and self-efficacy have both predicted youth wellbeing in past studies (Caprara et al., 2006; Park and Peterson, 2006). Research on a new parental style, SBP, indicates that when parents help teens to know about and use their strengths this also fosters adolescent wellbeing and academic outcomes (Waters, 2015b; Jach et al., 2017; Waters et al., in press). Increased self-efficacy is one promising mechanism by which SBP may promote wellbeing. As such, the current study confirmed early findings connecting SBP to teen wellbeing, and further tested the proposition that self-efficacy mediates the effect of SBP on teen mental health (happiness and distress) in a large sample of teenagers.

We hypothesized that (1) higher SBP would predict lower distress and higher happiness; (2) that self-efficacy would satisfy the general preconditions for mediation (Baron and Kenny, 1986) in that it would be related to SBP, distress and happiness; (3) self-efficacy would partially mediate relationships between SBP and both distress and happiness; and, after review, to explore whether any gender differences are apparent in the key findings. Results supported the hypotheses. Zero order correlations indicate SBP was inversely related to each component of distress, and positively related to happiness (**Table 1**). In addition, SBP, distress and happiness were all significantly related to self-efficacy. Proceeding to the mediation analysis, and after the stress measure was dropped mainly due to poor reliability, the final model indicated significant direct and indirect effects of SBP on the outcomes (**Figure 2**), and a very good fit to the data. Higher SBP predicted higher self-efficacy, and in-turn, lower depression and anxiety, and higher happiness. Importantly, this relationship did not nullify the significance of SBP’s direct relationship to both distress and happiness, and as such partial mediation was evident.

Finally, an exploratory multigroup analysis indicated no major differences in the solution across gender; especially in the effects of central interest. Lending support to the measures, only 3 of 17 items indicated factor loading invariance. Comparison of parameter estimates and fit indicators across nested models in which structural parameters were constrained to be equal suggested that the hypothesized model is a good representation of data for both males and females. Variance explained in the outcomes was slightly higher for males, and the effect of SBP on self-efficacy slightly larger for females. However, the key pathways of interest, i.e., the direct and indirect effects of SBP to the outcome variables, via self-efficacy, remained very similar across gender, with no changes in significance or sign of parameters, and minimal differences in magnitude.

The results of this study align with the past adaptive benefits of SBP, suggesting it promotes adolescent happiness while simultaneously acting as a protective factor against distress. In the present study with over 10,000 teenagers, results confirmed a direct and positive effect of SBP on higher happiness (β = 0.38), and a substantial inverse effects on depression (β = -0.45); but no direct effect on anxiety (β = 0.03). Specifically, these results indicate that with a 1 unit increase in standard deviation of SBP, there is a 0.38 increase in happiness, and a 0.45 decrease in depression (also in standard deviation units). In conjunction with self-efficacy, the model explained 56 and 40% of the variance in happiness and depression, respectively.

According to meta-analytically derived contemporary guidelines on effect sizes in individual difference research (Peterson and Brown, 2005; Gignac and Szodorai, 2016 support comparability), the direct effect of SBP on both happiness and distress is *large*, falling into the 85th and 95th percentiles of reported effect sizes, respectively (p. 75). These results are consistent with other studies on SBP which have found similarly large effect sizes. The total effect of SBP on teen stress was also large (β = -0.62) and explained 47% of stress variance (Waters, 2015a). SBP also uniquely predicted life satisfaction (Waters, 2015b), demonstrating a larger effect size (β = 0.41 for SBP use and β = 0.14 for SBP knowledge) and marginally larger portion of variance explained (adjusted *R*^2^ = 19%), than authoritative parenting styles (β = 0.16 and adjusted *R*^2^ = 17%). More recently, SBP uniquely predicted SWB, even after accounting for personality (β = 0.24), and with marginally greater benefit for those with growth mindsets (Jach et al., 2017).

The effects in the present study are also larger than some meta-analytic estimations of the average effect sizes for parental attachment on youth outcomes. As already noted, Mattanah et al. (2011) found a mean effect of *r* = 0.29 for parental attachment on positive development, and *r* = -0.21 for negative emotions, in college students. Similarly both the effect sizes of individual pathways in the model, and the variance explained in the current study in depression and anxiety, are both larger than meta-analytic derivations reported by McLeod et al. (2007a,b). Taken together, the generally large effect sizes for SBP on a diverse range of wellbeing indicators, falling on what can be described as both the positive and negative ends of the wellbeing spectrum (Keyes, 2002), place it amongst other important parental factors warranting further research. Indeed, it may be that SBP plays a bigger role than other styles of parenting such as attachment-oriented parenting and authoritative parenting. If SBP can be systematically increased, these findings also indicate that SBP is an important lever by which to increase teen wellbeing. At its broadest level, findings reiterate the importance of considering families rather than merely individuals in studies of wellbeing, and the often close connection between child and parent psychological factors (Williams et al., 2012).

The present study adds to the literature on process variables that connect SBP with adaptive outcomes. Prior studies have already identified some of the mechanisms by which SBP conveys effects. Jach et al. (2017) found that teen strength use accounted for 61.8% of the total effect of SBP on SWB, and Waters (2015a) found that teens own strength-based coping explained 29.3% of the total effect of SBP on reduced stress. The present study identified an additional important mechanism: global self-efficacy. Self-efficacy was found to partially and significantly mediate the effects of SBP on both happiness and distress factors. As a proportion of the standardized regression coefficients in **Figure 2**, the indirect effect of self-efficacy was substantial, accounting for 40.0 and 52.7% of the total effect of SBP on happiness and depression, respectively.

Interestingly, self-efficacy was a full mediator of the effect of SBP on anxiety. The difference between anxiety and depression here illustrates the uniqueness of these two distress factors, and how bundling them together may mask unique, nuanced findings. While SBP had no direct effect on anxiety, SBP boosted self-efficacy which in-turn reduced anxiety indirectly. This indirect effect was also large, with a standardized regression weight of similar magnitude to the indirect effect of SBP on depression. This finding also highlights the importance of investigating boundary effects, indicating self-efficacy is central to the benefits conveyed to teens in reducing anxiety.

Several theoretical explanations have been put forward for the links between SBP and teen wellbeing. They broadly revolve around education, reflection and reinforcement via stable and consistent feedback on strengths knowledge and use. Firstly, parents may educate children about their strengths, building their knowledge directly. Secondly, parents may reify their children’s own existing strengths schemas through social verification (Waters, 2015b), echoing and encouraging strengths processes in their children when they are visible. Thirdly, parents support strengths use by role modeling strengths use in themselves (Waters, 2015a), and also communicating and affirming an image of their child’s ‘ideal self,’ which they may then strive toward using their strengths (Waters, 2015b). The finding that SBP increases teen’s own strength use and children’s strength-based coping, with downstream benefits to wellbeing, is logical (see Waters, 2015a,b). Teen strength use is directly proximal to the SBP construct. But why would self-efficacy be promoted through SBP?

Connections between SBP and self-efficacy are somewhat more distal than the direct promotion of strengths knowledge and use in teens, but also theoretically plausible and call into consideration aspects of competency, causal and control beliefs in SBP and wellbeing research. As strengths are experienced as authentic and energizing (Govindji and Linley, 2007), increased strengths knowledge and use are likely to be highly adaptive in life. They may facilitate more accurate judgments of outcome expectancies and the matching of one’s abilities to external challenges and contexts, which would in-turn facilitate intrinsic motivation and engagement, goal-setting and striving (Zimmerman, 2000). Put more simply, teens with parents who build their strengths knowledge and use are likely to have higher general self-efficacy because they have learnt how to use their strengths to have agency over the actions and relationships. We theorize here that self-efficacy is the result of teens increasing their strengths knowledge and use via SBP, although longitudinal data is required to rule out the reverse, or a mutually reinforcing relationship.

The connections between self-efficacy, happiness and distress factors in the present study are also sensible and are consistent with past research showing a link between self-efficacy and wellbeing. Self-efficacy has been classed as a basic human need (Ryan and Deci, 2000; Bandura, 2008), and a form of eudaimonic happiness (Kashdan et al., 2008). This drive, intimately tied to environmental mastery, is likely central to survival and fostered through evolution. As humans have an inherent need to be effective, including meeting their goals, influencing their environment, and ultimately having a sense of stewardship over their lives, it is logical that those with higher self-efficacy would also have higher wellbeing. Happiness, as we define it, includes a component of hedonic happiness and an estimation of life satisfaction, placing this research in the general category of subjective wellbeing determinants.

### Limitations and Future Directions

This study had several strengths in its design and sample. Measuring both happiness and distress addressed calls from some researchers to study both the more well-established indicators of pathology/illbeing; as well as the more recently developed constructs/positive indicators of wellbeing, especially happiness (Wong, 2011). With over ten thousand teens in the data set, we had a large sample in this study, extending far beyond the sample sizes in prior strengths research, and larger even than most studies of teen wellbeing. Yet there are also several weaknesses.

Limitations of the present study included nascent validity for some of the measures, the lack of inclusion of prior process factors, and the inability to account for nested structures in the data. Also as a result of this study being undertaken within a larger measurement program, there were space limitations in the survey battery and hence some factors were only measured using two indicators.

That said, reliability and measurement model characteristics were acceptable according to guidelines, after removal of the stress subcomponent, and the content validity of the items was high given they were adapted from very well-established measures and adapted primarily for developmental appropriateness. Factor loadings were high. Tests of factor loading invariance indicated the great majority of items across gender did not systematically differ in interpretation, although the three items that did indicate there may be gendered interpretations of the words ‘cheerful’ and ‘fun,’ in relation to happiness; and also differences in attributions of effort to achieving desired outcomes.

Finally, with teenagers nested in schools that are certain to have distinct characteristics, multi-level modeling may have been able to better account for the nested structures in the data. However, due to ethical protocols established that anonymize schools from which the data was collected, we did not have indicators in this dataset by which to test for effects of group membership. Likewise, we were not able to report more detailed school characteristics such as socio-economic status, size or geographic location.

## Conclusion

Recent studies have shown that children and teens with strength-based parents experience less stress, enjoy life more, have higher wellbeing and do better academically speaking; this occurs through using their own strengths, engaging in strength-based coping, as well as having higher levels of engagement and perseverance. The benefits of SBP are also marginally greater for teenagers who see strengths as something that can be willfully changed. We can now add self-efficacy theoretical models connecting SBP to higher wellbeing outcomes. Future research may consider the role of SBP in addressing non-cognitive developmental disparities. These disparities are evident across socio-economic status in both academic and non-cognitive skills, including self-efficacy, and can impact life trajectories (Choi et al., 2016). When parents build strengths in their teenage children, they are also concurrently building self-efficacy, and maybe, thus, providing some of the knowledge and insight Twain spoke of in the opening quote that unlocks one’s ability and provides a pathway to wellbeing.

## Author Note

During creation of this manuscript the last author’s position was funded by the Gerry Higgins foundation. LW is also launching a book on strength-based parenting through Penguin Press: The Strength Switch. LW, Ph.D., has been an invited Keynote speaker on this topic at the 2nd Positive Education Schools Association Conference (pro-bono), the 3rd Canadian Positive Psychology Conference, (speaker honorarium) and the Festival of Positive Education (pro-bono). Lea is also co-founder of The Strengths Exchange, a website offering free strength-based resources and tools to parents (http://www.the-strengths-exchange.com.au) and the Strength Switch website (http://www.strengthswitch.com) another site that offers parents free and a paid for on-line strength-based parenting course.

## Ethics Statement

This study was carried out in accordance with the recommendations of the National Statement on Ethical Conduct in Human Research. Standing informed consent was provided by the parents of students who took part, and students were also given active assent to withdraw from the study. Their assent was confirmed via completion of the survey (rather than the collection of signed consent forms). The Principal of each school also provided consent for the school to participate. These protocols were approved by The University of Melbourne Humanities and Applied Sciences Human Research Ethics Committee (application number 1143313.1).

## Author Contributions

DL assisted in the conceptualization of the study, authored the introduction and discussion sections, handled the formatting and referencing, and refined and finalized the analysis and results. LW led the conceptualization of the study, and reviewed and edited all sections of the paper.

## Conflict of Interest Statement

The authors declare that the research was conducted in the absence of any commercial or financial relationships that could be construed as a potential conflict of interest.

## Footnotes

1 Happiness can be defined in many ways but a distinction has arisen in research between two broad types: ‘hedonic’ and ‘eudemonic’ (Kashdan et al., 2008; Huta and Waterman, 2014). These categories fall under the objectivist and subjectivist philosophical traditions, respectively, and are also sometimes referred to as ‘subjective wellbeing’ (hedonic) and ‘psychological wellbeing’ (eudaimonic). Hedonic happiness is generally experienced as a predominance of positive emotions, subjectively self-assessed over a given period of time. Eudemonic happiness is more cognitive and relates to a sense of meaning and purpose in life, generally through the pursuit of growth consistent with internal goals, a sense of self and code of ethics and virtues (Ryff and Singer, 2013).

2 The survey is known as the Wellbeing Profiler and can be accessed here: https://www.wbprofiler.com/

3 As the ethical protocols governing this larger program of school-based wellbeing measures were necessarily strict, only the variables of interest were extracted and analyzed for the purposes of this study. As such, the actual schools from which individual students were drawn remain anonymous and we were not able to report school characteristics or test for potential nested structures, including varied socioeconomic levels, in the data.

4 The second author of the current study was a member of the research team that initially developed the larger Wellbeing Profiler survey, and chose to include parent items so as to be able to address research questions and hypotheses of interest to her program of research on SBP.
